# Demography, commonly recorded disorders and mortality in chelonia under UK primary veterinary care: A VetCompass study

**DOI:** 10.1371/journal.pone.0321038

**Published:** 2025-04-09

**Authors:** Jessica May Hornby, Joanna Hedley, Dave C. Brodbelt, David B. Church, Dan G. O’Neill

**Affiliations:** 1 Beaumont Sainsbury Animal Hospital, The Royal Veterinary College, 4 Royal College Street, London, United Kingdom; 2 Pathobiology and Population Sciences, The Royal Veterinary College, Hawkshead Lane, North Mymms, Hatfield, Herts, United Kingdom; 3 Clinical Science and Services, The Royal Veterinary College, Hawkshead Lane, North Mymms, Hatfield, Herts, United Kingdom; University of Basrah, IRAQ

## Abstract

Little information has been published on the recorded disorders of pet chelonia. This cohort study aimed to report on demography, commonly recorded disorders and mortality recorded in chelonia under UK primary veterinary care in 2019. Electronic health records on a random sample of chelonia in VetCompass during 2019 were reviewed to extract species, recorded disorder and mortality data. Of 2,040 chelonia reviewed, 1,923 (94.26%) were recorded as tortoises, 74 (3.63%) terrapins and 43 (2.11%) turtles. Of the 811 (42.17%) tortoises with species information recorded, the most common species were Hermann’s tortoises (*Testudo hermanni*) (311, 38.35%), Horsfield’s tortoises (*Testudo horsfieldii*) (259, 31.94%) and Mediterranean spur-thighed tortoises (154, 18.99%). The most commonly recorded disorders were beak abnormalities (16.72%), overgrown nails (11.47%) and shell abnormalities (9.80%). The most common recorded causes of death were “disorder undiagnosed” (44.55%), dog bite (5.45%) and anorexia (3.96%). Of 178 deaths with age recorded, the median age at death was 7.32 years (IQR 2.50-15.14). Short temporal windows within the clinical records and often limited clinical workups were a frequent limitation to the precision of the recorded disorder terms. This is the largest study of chelonia to date. It has highlighted the most commonly recorded disorders of chelonia of which many are often linked to husbandry.

## Introduction

The order Chelonia, also referred to as Testudines or Testudinata, sits within the class of Reptilia and consists of 363 species [1]. The order Chelonia is commonly separated into the three groups using vernacular names of tortoises, terrapins and turtles. The interpretation for each vernacular term varies around the world but within the United Kingdom (UK), tortoise generally refers to terrestrial species, terrapin to freshwater species and turtle to marine chelonia [2]. However, certain vernacular names still contradict their classification. For example, *Sternotherus odoratus* is known as the common musk turtle but lives in/around freshwater rather than in a marine environment [3].

Chelonia represent the eighth most common group of companion animals in the UK, with 1.8% of UK households estimated to own at least one tortoise or terrapin [4]. A 2022 survey reported the most kept species of tortoises in the UK were Hermann’s tortoise (*Testudo hermanni*), and the most kept genus of terrapins were musk turtles (*Sternotherus spp*.) [5]. However, limited demographic data exist on UK and worldwide chelonia for several reasons. Most national data collection on wild and captive animal population numbers parallels conservation studies but reptiles have been largely overlooked in much of the conservation prioritisation [6]. Trade data offer one resource on population numbers, but such data can be extremely difficult to attain, with true data completeness further complicated by illegal trade [7]. International movement of animal species classified as endangered is regulated by the Convention on International Trade in Endangered Species (CITES) legislation [8]. A 2021 report revealed that 245,273 live vertebrate CITES-listed species entered the UK from 2010 to 2019 and that, of these, 86.7%, (n = 212,666) were reptiles; Mediterranean tortoises were the most frequently imported reptile species (56.3%, n = 119,634) [9]. Regarding non-CITES listed wildlife, a 2020 study reported reptiles as the second most common category of animals imported (16.8%, n =  578,772) into the UK between 2014 and 2018, with Testudinata comprising 38.74% (n =  224,237) of these imported reptiles (note: excluding Columbiformes and Galliformes which made up the vast majority (n =  45,518,548) [10]. These reports suggest large numbers of chelonia are imported legally into the UK. Despite limited data on illegal imports due to the nefarious nature of this activity, criminal records do support that illegal importation occurs [11]. Due to these illegal imports it is impossible to give exact total figures. Establishing better data on which chelonian species are kept as pets in the UK would guide veterinary professionals to improve their knowledge on these species.

In chelonia specifically, prevalence for specific disorders has been reported in certain groups of animals. A retrospective study of fibropapillomatosis reported increasing prevalence in wild green sea turtles (*Chelonia mydas*) over time, while a prospective study of gastrointestinal parasitic burdens reported 49% of captive tortoises with at least one parasite [12,13]. However, there is very limited evidence on the most common disorders of pet chelonia overall in the UK. A study from 1979 reviewed clinical records of 70 tortoises from a single UK veterinary clinic to report the top three disorders as gastrointestinal helminthiasis, necrotic stomatitis and hypovitaminosis but that study was limited by both small sample size, selection bias and also by limited work-up for diagnoses for example hypovitaminosis was diagnosed based on clinical signs alone [14]. However, it is also important to acknowledge that these limitations from a research perspective often represent the reality of primary care practice, i.e., not performing liver biopsies to diagnose hypovitaminosis [15], and it is still important to understand the primary care view given that this is where the majority of pet species acquire their veterinary care. A stronger evidence base on the current health issues of pet chelonia and of the primary veterinary care given to this species can contribute to tailored further education, practical experience and purchasing equipment to assist veterinary professionals provide improved clinical care and ultimately improve animal welfare.

There are very limited longevity and mortality data on chelonia owned in the UK. Chelonia are reported as amongst the longest-lived reptiles but accurately aging these animals once they reach adulthood is challenging [16]. Some limited research is published on longevity of reptiles in zoological collections [17]. A recent UK survey reported a low mortality of reptiles at home [18]. To the authors’ knowledge, no further studies have been published on mortality of chelonia kept in the UK although such longevity data are critical to contribute to welfare assessment.

With a perspective of providing evidence that can generalise to the wider population of UK chelonia, this study aimed to report the frequencies of common disorders along with demographic and mortality data on the UK chelonia population under primary veterinary care during 2019. These results could assist veterinary professionals and owners with an evidence base to better understand and predict likely disorder occurrence and to identify key health and welfare opportunities for chelonia.

## Methods

The study population included all chelonia under primary veterinary care at clinics participating in the VetCompass Programme during 2019. To be included within the study, chelonia under veterinary care required at least one electronic health patient record (EHR; free-text clinical note or treatment) recorded during 2019. VetCompass collates de-identified EHR data from veterinary practices in the UK for epidemiological research [19]. The veterinary practices provide written consent and the animal owners provide verbal opt out consent. Relevant data fields available to VetCompass researchers include a unique animal identifier along with veterinary group identifier, species, date of birth and sex along with clinical information from free-form text clinical notes and treatment with relevant dates. In the UK, corporates, termed “veterinary groups” in this paper, run many individual veterinary practices.

A cohort study design was used to estimate the one-year (2019) period prevalence of the most commonly diagnosed disorders. Sample size calculations estimated that at least 1,814 chelonia were needed to report the prevalence for a disorder occurring in 5.0% of chelonia with 1.0% margin of error at a 95% confidence level, assuming a UK population of 300,000 chelonia [4,20]. Ethics approval was obtained from the RVC Ethics and Welfare Committee prior to the study commencing (reference number SR2018-1652).

A random sample of 2,040 chelonia was selected from an overall sampling frame of 4,432 chelonia under veterinary care in 2019. These chelonia were randomly ordered and all information in the EHR relating to 2019 was reviewed manually between 11^th^ February 2022 and 1^st^ June 2023 to extract the most definitive final recorded disorders (herein after shown as ‘recorded disorders’) recorded in the EHR as existing during 2019 as previously described [21]. The EHRs were accessed in the VetCompass online user interface (VetCompass.org). Elective (e.g., microchipping) or prophylactic (e.g., parasiticide therapy without faecal analysis) clinical events were not included. No distinction was made between pre-existing and incident disorder presentations. When a final biomedical diagnosis was not made, the clinical sign that summarised the clinical phenotype of the disorder most appropriately was accepted for the current study, e.g., “anorexia” was accepted if a more formal biomedical aetiology was not achieved to explain the anorexia. This approach aimed to present an accurate reflection of the overall caseload and the nature of the disorders that are recorded in routine primary veterinary care in the UK. All extracted disorders were mapped to a dual hierarchy of diagnostic precision for analysis: precise-level precision and grouped-level precision as previously described [22]. Briefly, precise-level precision terms described the original extracted terms at the maximal diagnostic precision recorded within the clinical notes (e.g., *dystocia* would remain as *dystocia*). Grouped-level precision terms mapped the original disorder terms to a general level of diagnostic precision (e.g., *dystocia* would map to *female reproductive disorder*).

Demographic data were extracted for each chelonian that combined the information in the formal demographic data fields with any supplemental information recorded in the free text clinical notes. Species names were defined in accordance with Reptile Database [23] and recorded using the most precise term supported in the overall EHR. For example, if ‘tortoise’ was the only species information recorded in the formal species field but if ‘*Testudo hermanni’* was recorded in the free text clinical notes, the chelonian was recorded as tortoise and the species as Hermann’s Tortoise. Vernacular terms were extracted using English definitions as discussed above. Chelonia recorded as spur-thighed tortoises were grouped with those listed as Mediterranean spur-thighed tortoises (*Testudo graeca*) and not Sulcata tortoises (*Centrochelys sulcata*) because Sulcata tortoises are often referred to as African spurred tortoises rather than spur-thighed [24]. Mortality data on the method (euthanasia, unassisted death, unrecorded), date of death and biomedical cause of death were extracted for all deaths that occurred at any date in the available EHRs.

Following data checking for internal validity and cleaning in Excel (Microsoft Office Excel, Microsoft Corp.), analyses were conducted using SPSS version 29.0 (IBM Corp). Age (years) was defined on December 31, 2019, as the final date by which each chelonian was classified as either a case or a non-case for each disorder. Continuous data were checked for normal distribution and described using the mean (standard deviation) for normally distributed data and the median (interquartile range, range) for non-normally distributed data [25].

One-year period prevalence values with 95% confidence intervals (CI) described the probability of a disorder at least once during 2019. The term period prevalence was defined as the proportion of the denominator population that were diagnosed with the disorder at least once during the specified time. Confidence interval estimates were derived from standard errors based on approximation to the binomial distribution [25]. Prevalence values were reported for chelonia overall and separately for tortoises, terrapins and turtles, and also for the top three tortoise and top two terrapin species. Univariable comparisons compared categorical variables using the chi-squared test unless at least 20% of values had an expected count <  5 when the Fisher’s exact test was used [25, 26]. Statistical significance was set at the 5% level.

## Results

### Demography

Chelonia comprised 4,432/3,885,917 (0.11%) of all animals under primary veterinary care across 6 veterinary groups in VetCompass during 2019. From the 4,432 available chelonia, the current analysis included a random sample of 2,040 (46.03%) chelonia recorded as 1923 (94.26%) tortoises, 74 (3.63%) terrapins and 43 (2.11%) turtles. Because the study did not involve any living animals whose welfare might be impinged by including more animals in the study beyond the minimum required, it was decided to include more animals than necessary by the sample size estimate. Most records (n =  1192, 58.43%) did not specify the species of the chelonian. Among the 848 (41.57%) chelonia with species information available, 24 different species were recorded. The most common tortoise species were Hermann’s tortoise (*Testudo hermanni*) (n =  311, 15.25%), Horsfield’s tortoise (*Testudo horsfieldii*) (259, n =  12.70%) and Mediterranean spur-thighed tortoise (*Testudo graeca*) (n =  154, 7.55%). The most common terrapin species were musk turtle (*Sternotherus spp.*) (n =  17, 0.83%) and yellowbelly slider (*Trachemys scripta scripta*) (n =  9, 0.44%). No marine turtle species were recorded (Table 1).

**Table 1:** Numbers of chelonia with information available on species under primary veterinary care at practices participating in the VetCompass™ Programme in the United Kingdom from January 1 to December 31, 2019. Note: some chelonia were recorded only by genus. N =  2,040.

Chelonia	No.	% of all chelonia (n = 2040)
Species unrecorded	1192	58.43
**Tortoise species**	**No.**	**% of chelonia with species recorded (n = 848)**
Hermann’s Tortoise (*Testudo hermanni*)	311	36.67
Horsfield’s Tortoise (*Testudo horsfieldii*)	259	30.54
Mediterranean Spur-thighed Tortoise (*Testudo graeca*)	154	18.16
Leopard Tortoise (*Stigmochelys pardalis*)	23	2.71
Marginated Tortoise (*Testudo marginata*)	22	2.59
Sulcata Tortoise (*Centrochelys sulcata*)	17	2.00
Red-footed Tortoise (*Chelonoidis carbonarius*)	12	1.42
Indian Star Tortoise (*Geochelone elegans*)	4	0.47
Pancake Tortoise (*Malacochersus tornieri*)	3	0.35
Elongated Tortoise (*Indotestudo elongata*)	1	0.12
Egyptian Tortoise (*Testudo kleinmanni*)	1	0.12
Bolson Tortoise (*Gopherus flavomarginatus*)	1	0.12
Desert Tortoise (*Gopherus agassizii*)	1	0.12
Hingeback Tortoise (*Kinixys spp.*)	1	0.12
Bell’s Hingeback Tortoise (*Kinixys zombensis*)	1	0.12
**Terrapin species**		
Musk Turtle (*Sternotherus spp.*)	17	2.00
Yellowbelly Slider (*Trachemys scripta scripta*)	9	1.06
Razorback Musk Turtle (*Sternotherus carinatus*)	3	0.35
Box Turtle (*Terrapene spp.*)	3	0.35
Cooter (*Pseudemys spp.*)	1	0.12
Red-eared Slider (*Trachemys scripta elegans*)	1	0.12
Loggerhead Musk Turtle (*Sternotherus minor*)	1	0.12
European Pond Turtle (*Emys orbicularis*)	1	0.12
Common Snapping Turtle (Chelydra serpentina)	1	0.12
Total	2040	100%

Of the 2,040 chelonians, 1,047 (51.32%) were recorded as male, 759 (37.21%) as female, with sex unrecorded in 234 (11.47%). Of the 2,040 chelonians, the median age was 9.31 years (IQR: 3.99 to 25.40, range: 0.03 to 120.67). Median age of tortoises was 9.41 years (IQR: 4.05 to 25.34, range: 0.03 to 120.67), of terrapins was 7.57 years (IQR: 3.13 to 20.12, range: 0.57 to 120.00) and of turtles was 7.58 years (IQR: 1.59 to 120.00, range: 0.77 to 120.00).

### Recorded disorder prevalence

Overall, 1595/2040 (78.19%) chelonia had at least one recorded disorder during 2019. Vernacular chelonian group was significantly associated with the probability of animals being recording with at least one disorder (p = 0.029). Across the three groups, tortoise had the lowest probability of being recorded with at least one disorder recorded (1492/1923, 77.59%), followed by terrapin 65/74 (87.84%) and turtle 38/43 (88.37%). Of the 2040 chelonia, the median number of recorded disorders per chelonian overall was one (IQR: 1 to 1, range: 0 to 7). For tortoises the median number of recorded disorders per animal was one (IQR: 1 to 1, range: 0 to 7). For terrapins the median number of recorded disorders per animal was one (IQR: 1 to 2, range: 0 to 4). Finally, for turtles the median number of recorded disorders per animal was one (IQR: 1 to 1, range: 0 to 3).

Across all 2040 chelonia, 2272 unique disorder events were recorded that encompassed 230 precise-level terms and 62 group-level terms. The most common precise-level disorders overall included beak abnormality, overgrown nail(s), shell abnormality and anorexia. A statistically significant difference in prevalence was detected between tortoises, terrapins and turtles for 2 from among the 4 most common disorders at precise level in chelonia (Table 2).

**Table 2:** Prevalence of the most common recorded disorders at a precise level of diagnostic precision recorded in all chelonia (n = 2040) under primary-care veterinary care at UK practices participating in the VetCompass™ Programme from January 1 to December 31, 2019. *  CI confidence interval ** P-value for association between vernacular chelonian group (tortoise, terrapin and turtle) and the disorder.

Precise-level term	All chelonia (n = 2040) No. (%)	95% CI *	Tortoise (n = 1923) No. (%)	Terrapin (n = 74) No. (%)	Turtle (n = 43)No. (%)	P-value**
Beak abnormality	341 (16.72)	15.16-18.40	340 (17.68)	0 (0.00)	1 (2.33)	<0.001
Overgrown nail(s)	234 (11.47)	10.16-12.93	229 (11.91)	4 (5.41)	1 (2.33)	0.037
Shell abnormality	200 (9.80)	8.59-11.17	186 (9.67)	10 (13.51)	4 (9.30)	0.548
Anorexia	95 (4.66)	3.82-5.66	89 (4.63)	3 (4.05)	3 (6.98)	0.671
Disorder undiagnosed	81 (3.97)	3.21-4.91	67 (3.48)	10 (13.51)	4 (9.30)	<0.001
Ophthalmological disorder	72 (3.53)	2.81-4.42	66 (3.43)	4 (5.41)	2 (4.65)	0.401
Dog bite	49 (2.40)	1.82-3.16	48 (2.50)	0 (0.00)	1 (2.33)	0.524
Nasal discharge	49 (2.40)	1.82-3.16	49 (2.55)	0 (0.00)	0 (0.00)	0.290
Dystocia	42 (2.06)	1.53-2.77	41 (2.13)	1 (1.35)	0 (0.00)	> 0.999
Upper respiratory tract infection	41 (2.01)	1.48-2.72	39 (2.02)	2 (2.70)	0 (0.00)	0.730
Inappetence	33 (1.62)	1.15-2.26	33 (1.72)	0 (0.00)	0 (0.00)	0.816
Plastron abnormal	31 (1.52)	1.07-2.15	25 (1.30)	6 (8.11)	0 (0.00)	0.001
Carapace abnormal	30 (1.47)	1.03-2.09	29 (1.51)	0 (0.00)	1 (2.33)	0.509
Lethargy	29 (1.42)	0.99-2.03	26 (1.35)	3 (4.05)	0 (0.00)	0.160
Pinworms	29 (1.42)	0.99-2.03	29 (1.51)	0 (0.00)	0 (0.00)	0.786
Metabolic bone disease	27 (1.32)	0.91-1.92	25 (1.30)	1 (1.35)	1 (2.33)	0.591
Respiratory noise increased	27 (1.32)	0.91-1.92	25 (1.30)	2 (2.70)	0 (0.00)	0.341
Wound	27 (1.32)	0.91-1.92	23 (1.20)	1 (1.35)	3 (6.98)	0.022
Parasite infestation	24 (1.18)	0.79-1.74	24 (1.25)	0 (0.00)	0 (0.00)	> 0.999
Cloacal prolapse	23 (1.13)	0.75-1.69	21 (1.09)	2 (2.70)	0 (0.00)	0.267

For tortoises (n =  1923), the most common precise-level recorded disorders were beak abnormality (n = 340, prevalence 17.68%, 95% CI: 16.04–19.45), overgrown nails (n = 229, prevalence 11.91%, 95% CI: 10.54-13.43) and shell abnormality (n = 186, prevalence 9.67%, 95% CI: 8.43-11.08). For terrapins (n =  74), the most common precise-level disorders were disorder undiagnosed (n = 10, prevalence 13.51%, 95% CI: 7.51-23.12), shell abnormality (n = 10, prevalence 13.51%, 95% CI: 7.51-23.12) and plastron abnormality (n = 6, prevalence 8.11%, 95% CI: 3.77-16.58). For turtles (n =  43), the most common precise level disorders were vitamin A deficiency (n = 4, prevalence 9.3%, 95% CI: 3.68-21.60), disorder undiagnosed (n = 4, prevalence 9.3%, 95% CI: 3.68-21.60) and shell abnormality (n = 4, prevalence 9.3%, 95% CI: 3.68-21.60). Table 3 lists the most common disorders of the most common species.

**Table 3:** Prevalence of the most common recorded disorders at a precise level of diagnostic precision recorded in the three most common tortoise and two most common terrapin genus/species under primary-care veterinary care at UK practices participating in the VetCompass™ Programme from January 1 to December 31, 2019.

Precise-level term	Frequency	Percentage	95% CI
**Hermann’s tortoise (n = 311)**			
Beak abnormality	63	20.26	16.17–25.07
Shell abnormality	50	16.08	12.41–20.57
Overgrown nails	19	6.11	3.95–9.34
**Horsfield’s tortoise (n = 259)**			
Beak abnormality	54	20.85	16.35–26.20
Overgrown nails	31	11.97	8.56–16.49
Shell abnormality	31	11.97	8.56–16.49
**Mediterranean Spur–thighed tortoise (n = 154)**			
Shell abnormality	15	9.74	5.99–15.45
Beak abnormality	11	7.14	4.03–12.34
Overgrown nails	11	7.14	4.03–12.34
**Musk turtles (n = 17)**			
Shell abnormality	3	17.65	6.19–41.03
Disorder undiagnosed	2	11.76	3.29–34.34
Plastron abnormality	2	11.76	3.29–34.34
**Yellowbelly sliders (n = 9)**			
Shell abnormality	3	33.33	12.06–64.58
Disorder undiagnosed	2	22.22	6.32–54.74

The most common grouped-level recorded disorders included beak disorder, skin disorder, claw/nail disorder and respiratory tract disorder. A statistically significant difference in prevalence was detected between tortoises, terrapins and turtles for 2 from among the 4 most common disorders at group level in chelonia (Table 4).

**Table 4:** Prevalence of the most common recorded disorders at a grouped level of diagnostic precision was recorded in all chelonia (n = 2040) under primary-care veterinary care at UK practices participating in the VetCompass™ Programme from January 1 to December 31, 2019. *  CI confidence interval ** P-value for association between vernacular chelonian group (tortoise, terrapin and turtle) and the disorder.

Grouped-level term	All chelonia (n = 2040) No. (%)	95% CI ^*^	Tortoise (n = 1923) No. (%)	Terrapin (n = 74) No. (%)	Turtle (n = 43) No. (%)	P-value^**^
Beak disorder	341 (16.72)	15.16–18.40	340 (17.68)	0 (0.00)	1 (2.33)	<0.001
Skin disorder	301 (14.75)	13.28–16.36	276 (14.35)	18 (24.32)	7 (16.28)	0.057
Claw/nail disorder	244 (11.96)	10.62–13.44	239 (12.43)	4 (5.41)	1 (2.33)	0.027
Respiratory tract disorder	174 (8.53)	7.39–9.82	163 (8.48)	8 (10.81)	3 (6.98)	0.729
Ophthalmological disorder	162 (7.94)	6.85–9.20	145 (7.54)	13 (17.57)	4 (9.30)	0.007
Traumatic injury	132 (6.47)	5.48–7.62	119 (6.19)	6 (8.11)	7 (16.28)	0.036
Appetite disorder	129 (6.32)	5.35–7.46	123 (6.40)	3 (4.05)	3 (6.98)	0.786
Parasite infestation	109 (5.34)	4.45–6.41	109 (5.67)	0 (0.00)	0 (0.00)	0.020
Disorder undiagnosed	81 (3.97)	3.21–4.91	67 (3.48)	10 (13.51)	4 (9.30)	<0.001
Female reproductive disorder	47 (2.30)	1.74–3.05	46 (2.39)	1 (1.35)	0 (0.00)	0.881
Musculoskeletal disorder	47 (2.30)	1.74–3.05	42 (2.18)	5 (6.76)	0 (0.00)	0.045
Lethargy	32 (1.57)	1.11–2.21	29 (1.51)	3 (4.05)	0 (0.00)	0.201
Enteropathy	31 (1.52)	1.07–2.15	30 (1.56)	1 (1.35)	0 (0.00)	> 0.999
Oral cavity disorder	30 (1.47)	1.03–2.09	28 (1.46)	2 (2.70)	0 (0.00)	0.385
Cloacal disorder	27 (1.32)	0.91–1.92	25 (1.30)	2 (2.70)	0 (0.00)	0.335
Metabolic bone disease	27 (1.32)	0.91–1.92	25 (1.30)	1 (1.35)	1 (2.33)	0.584
Mass	25 (1.23)	0.83–1.80	24 (1.25)	1 (1.35)	0 (0.00)	0.778
Thin	22 (1.08)	0.71–1.63	20 (1.04)	2 (2.70)	0 (0.00)	0.259
Congenital disorder	21 (1.03)	0.67–1.57	21 (1.09)	0 (0.00)	0 (0.00)	> 0.999
Foreign body	21 (1.03)	0.67–1.57	20 (1.04)	0 (0.00)	1 (2.33)	0.475

The most common grouped-level recorded disorders for tortoises (n =  1923) were beak disorder (n = 340, prevalence 17.68%, 95% CI: 16.04–19.45), skin disorder (n = 276, prevalence 14.35%, 95% CI: 12.86–15.99) and claw/nail disorder (n = 239, prevalence 12.43%, 95% CI: 11.03–13.98). The most common grouped-level disorders for terrapins (n =  74) were skin disorder (n = 18, prevalence 24.32%, 95% CI: 15.98–35.21), ophthalmological disorder (n = 13, prevalence 17.57%, 95% CI: 10.56–27.77) and disorder undiagnosed (n = 10, prevalence 13.51%, 95% CI: 7.51–23.12). The most common grouped level disorders or turtles (n =  43) were skin disorder (n = 7, prevalence 16.28%, 95% CI: 8.12–29.97) and traumatic injury (n = 7, prevalence 16.28%, 95% CI: 8.12–29.97). Table 5 lists the most common disorders of the most common species.

**Table 5:** Prevalence of the most common recorded disorders at a grouped level of diagnostic precision recorded in the three most common tortoise and two most common terrapin genus/species under primary-care veterinary care at UK practices participating in the VetCompass™ Programme from January 1 to December 31, 2019.

Grouped-level term	Frequency	Percentage	95% CI
**Hermann’s tortoise (n = 311)**			
Skin disorder	72	23.15	18.81–28.15
Beak disorder	63	20.26	16.17–25.07
Respiratory tract disorder	25	8.04	5.50–11.60
**Horsfield’s tortoise (n = 259)**			
Beak disorder	54	20.85	16.35–26.20
Skin disorder	47	18.15	13.93–23.29
Ophthalmological disorder	35	13.51	9.88–18.21
**Mediterranean Spur-thighed tortoise (n = 154)**			
Respiratory tract disorder	23	14.94	10.16–21.41
Skin disorder	19	12.34	8.04–18.47
**Musk turtles (n = 17)**			
Skin disorder	5	29.41	13.28–53.13
Respiratory tract disorder	3	17.65	6.19–41.03
Traumatic injury	3	17.65	6.19–41.03
**Yellowbelly sliders (n = 9)**			
Skin disorder	3	33.33	12.06–64.58
Musculoskeletal disorder	2	22.22	6.32–54.74
Disorder undiagnosed	2	22.22	6.32–54.74

### Mortality

Death was recorded in the clinical records for 202/2040 (9.90%) chelonians. Of these deaths, 172 (85.15%) were tortoises, 22 (10.89%) were terrapins and 8 (3.96%) were turtles. Of 178/202 (88.12%) deaths with the age at death available, the median age of death was 7.32 years (IQR 2.50–15.14, range: 0.00–113.45). Overall, 100 (49.50%) deaths were unassisted, 94 (46.53%) were euthanased and eight (3.96%) were unrecorded.

The most common causes of death at precise-level included disorder undiagnosed, dog bite and anorexia. A statistically significant difference in prevalence was not detected between tortoises, terrapins and turtles for the 3 most common causes of death at precise level in chelonia (Table 6).

**Table 6:** Mortality data from the most common recorded disorders at a precise level of diagnostic precision was recorded in all chelonia (n = 202) under primary-care veterinary care at UK practices participating in the VetCompass™ Programme from January 1 2019. *  CI confidence interval ** P-value for association between vernacular chelonian group (tortoise, terrapin and turtle) and the disorder.

Precise-level term	All chelonia (n = 202) No. (%)	95% CI *	Tortoise (n = 172) No. (%)	Terrapin (n = 22) No. (%)	Turtle (n = 8)No. (%)	P-value**
Disorder undiagnosed	90 (44.55)	37.87–51.45	76 (44.19)	10 (45.45)	4 (50.00)	> 0.999
Dog bite	11 (5.45)	3.07–9.49	11 (6.40)	0 (0.00)	0 (0.00)	0.757
Anorexia	8 (3.96)	2.02–7.62	5 (2.91)	3 (13.64)	0 (0.00)	0.080
Cloacal prolapse	7 (3.47)	1.69–6.98	7 (4.07)	0 (0.00)	0 (0.00)	> 0.999
Upper respiratory tract infection	7 (3.47)	1.69–6.98	6 (3.49)	1 (4.55)	0 (0.00)	0.683
Dystocia	5 (2.48)	1.06–5.66	5 (2.91)	0 (0.00)	0 (0.00)	> 0.999
Metabolic bone disease	5 (2.48)	1.06–5.66	5 (2.91)	0 (0.00)	0 (0.00)	> 0.999
Kidney failure	4 (1.98)	0.77–4.98	4 (2.33)	0 (0.00)	0 (0.00)	> 0.999
Sepsis	4 (1.98)	0.77–4.98	2 (1.16)	1 (4.55)	1 (12.50)	0.058
Collapsed	3 (1.49)	0.51–4.27	3 (1.74)	0 (0.00)	0 (0.00)	> 0.999
Dyspnoea	3 (1.49)	0.51–4.27	3 (1.74)	0 (0.00)	0 (0.00)	> 0.999
Ophthalmological disorder	3 (1.49)	0.51–4.27	3 (1.74)	0 (0.00)	0 (0.00)	> 0.999
Pneumonia	3 (1.49)	0.51–4.27	2 (1.16)	1 (4.55)	0 (0.00)	0.384
Traumatic injury	3 (1.49)	0.51–4.27	3 (1.74)	0 (0.00)	0 (0.00)	> 0.999
Animal bite	2 (0.99)	0.27–3.54	2 (1.16)	0 (0.00)	0 (0.00)	> 0.999
Azotaemia	2 (0.99)	0.27–3.54	2 (1.16)	0 (0.00)	0 (0.00)	> 0.999
Chronic kidney failure	2 (0.99)	0.27–3.54	2 (1.16)	0 (0.00)	0 (0.00)	> 0.999
Coelomic mass	2 (0.99)	0.27–3.54	0 (0.00)	2 (9.09)	0 (0.00)	0.023
Flystrike	2 (0.99)	0.27–3.54	2 (1.16)	0 (0.00)	0 (0.00)	> 0.999
Lethargy	2 (0.99)	0.27–3.54	2 (1.16)	0 (0.00)	0 (0.00)	> 0.999
Metabolic disorder	2 (0.99)	0.27–3.54	1 (0.58)	1 (4.55)	0 (0.00)	0.276
Respiratory noise increased	2 (0.99)	0.27–3.54	2 (1.16)	0 (0.00)	0 (0.00)	> 0.999

The most common causes of death at precise-level for tortoises were disorder undiagnosed (n = 76, prevalence 44.19%, 95% CI: 36.97-51.65), dog bite (n = 11, prevalence 6.40%, 95% CI: 3.61-11.09) and cloacal prolapse (n = 7, prevalence 4.07%, 95% CI: 1.99-8.16). The most common causes of death at precise-level for terrapins were disorder undiagnosed (n = 10, prevalence 45.45%, 95% CI: 26.92-65.34), anorexia (n = 3, prevalence 13.64%, 95% CI: 4.75-33.33) and coelomic mass (n = 2, prevalence 9.09%, 95% CI: 2.53-27.81). The most common causes of death at precise-level for turtles was disorder undiagnosed (n = 4, prevalence 50.00%, 95% CI: 21.52-78.48). Table 7 lists the most common disorders resulting in death of the most common species.

**Table 7:** Mortality data from the most common disorders at a precise level of diagnostic precision was recorded in the three most common tortoise and two most common terrapin genus/species under primary-care veterinary care at UK practices participating in the VetCompass™ Programme from January 1 2019.

Precise-level term	Frequency	Percentage	95% CI
**Hermann’s tortoise**			
Disorder undiagnosed	7	25.00	12.68–43.36
Dystocia	3	10.71	3.71–27.20
**Horsfield’s tortoise**			
Disorder undiagnosed	10	47.62	28.34–67.63
Upper respiratory tract infection	2	9.52	2.65–28.91
**Mediterranean Spur-thighed tortoise**			
Disorder undiagnosed	5	25.00	11.19–46.87
Kidney failure	2	10.00	2.79–30.10
Metabolic bone disease	2	10.00	2.79–30.10
**Musk turtles**			
Disorder undiagnosed	2	50.00	15.00–85.00
Ascites	1	25.00	4.56–69.94
Pneumonia	1	25.00	4.56–69.94
**Yellowbelly sliders**			
Disorder undiagnosed	2	100.00	34.24–100.00

The most common causes of death at grouped-level included disorder undiagnosed, respiratory tract disorder and traumatic injury. A statistically significant difference in prevalence was not detected between tortoises, terrapins and turtles for the 3 most common causes of death at group level in chelonia (Table 8).

**Table 8:** Mortality data from the most common disorders at a grouped level of diagnostic precision was recorded in all chelonia (n = 202) under primary-care veterinary care at UK practices participating in the VetCompass™ Programme from January 1 2019. *  CI confidence interval ** P-value for association between vernacular chelonian group (tortoise, terrapin and turtle) and the disorder.

Grouped-level term	All chelonia (n = 202) No. (%)	95% CI *	Tortoise (n = 172) No. (%)	Terrapin (n = 22) No. (%)	Turtle (n = 8)No. (%)	P-value**
Disorder undiagnosed	90 (44.55)	37.87–51.45	76 (44.19)	10 (45.45)	4 (50.00)	> 0.999
Respiratory tract disorder	17 (8.42)	5.32–13.06	14 (8.14)	2 (9.09)	1 (12.50)	0.604
Traumatic injury	17 (8.42)	5.32–13.06	17 (9.88)	0 (0.00)	0 (0.00)	0.332
Kidney disease	9 (4.46)	2.36–8.25	9 (5.23)	0 (0.00)	0 (0.00)	0.722
Appetite disorder	8 (3.96)	2.02–7.62	5 (2.91)	3 (13.64)	0 (0.00)	0.073
Cloacal disorder	7 (3.47)	1.69–6.98	7 (4.07)	0 (0.00)	0 (0.00)	> 0.999
Female reproductive disorder	7 (3.47)	1.69–6.98	7 (4.07)	0 (0.00)	0 (0.00)	> 0.999
Metabolic bone disease	5 (2.48)	1.06–5.66	5 (2.91)	0 (0.00)	0 (0.00)	> 0.999
Ophthalmological disorder	4 (1.98)	0.77–4.98	4 (2.33)	0 (0.00)	0 (0.00)	> 0.999
Sepsis	4 (1.98)	0.77–4.98	2 (1.16)	1 (4.55)	1 (12.50)	0.056
Collapsed	3 (1.49)	0.51–4.27	3 (1.74)	0 (0.00)	0 (0.00)	> 0.999
Lethargy	3 (1.49)	0.51–4.27	3 (1.74)	0 (0.00)	0 (0.00)	> 0.999
Mass	3 (1.49)	0.51–4.27	1 (0.58)	2 (9.09)	0 (0.00)	0.061
Abdominal disease	2 (0.99)	0.27–3.54	1 (0.58)	1 (4.55)	0 (0.00)	0.280
Brain disorder	2 (0.99)	0.27–3.54	1 (0.58)	1 (4.55)	0 (0.00)	0.274
Complication associated with clinical care	2 (0.99)	0.27–3.54	2 (1.16)	0 (0.00)	0 (0.00)	> 0.999
Hereditary metabolic disorder	2 (0.99)	0.27–3.54	1 (0.58)	1 (4.55)	0 (0.00)	0.268
Parasite infestation	2 (0.99)	0.27–3.54	2 (1.16)	0 (0.00)	0 (0.00)	> 0.999
Skin disorder	2 (0.99)	0.27–3.54	2 (1.16)	0 (0.00)	0 (0.00)	> 0.999
Urinary system disorder	2 (0.99)	0.27–3.54	2 (1.16)	0 (0.00)	0 (0.00)	> 0.999

The most common causes of death at grouped level for tortoises were disorder undiagnosed (n = 76, prevalence 44.19%, 95% CI: 36.97–51.65), traumatic injury (n = 17, prevalence 9.88%, 95% CI: 6.26–15.26) and respiratory tract disorder (n = 14, prevalence 8.14%, 95% CI: 4.91–13.20). The most common causes of death at grouped level for terrapins were disorder undiagnosed (n = 10, prevalence 45.45%, 95% CI: 26.92–65.34) and appetite disorder (n = 3, prevalence 13.64%, 95% CI: 4.75–33.33). The most common causes of death at grouped level for turtles was disorder undiagnosed (n = 4, prevalence 50.00%, 95% CI: 21.52–78.48). Table 9 lists the most common disorders resulting in death of the most common species.

**Table 9:** Mortality data from the most common disorders at a grouped level of diagnostic precision was recorded in the three most common tortoise and two most common terrapin genus/species under primary-care veterinary care at UK practices participating in the VetCompass™ Programme from January 1 2019.

Grouped-level term	Frequency	Percentage	95% CI
**Hermann’s tortoise**			
Disorder undiagnosed	7	25.00	12.68–43.36
Kidney disease	4	14.29	5.70–31.49
**Horsfield’s tortoise**			
Disorder undiagnosed	10	47.62	28.34–67.63
Respiratory tract disorder	4	19.05	7.67–40.00
Traumatic injury	2	9.52	2.65–28.91
**Mediterranean Spur-thighed tortoise**			
Disorder undiagnosed	5	25.00	11.19–46.87
**Musk turtles**			
Disorder undiagnosed	2	50.00	15.00–85.00
Abdominal disease	1	25.00	4.56–69.94
Respiratory tract disorder	1	25.00	4.56–69.94
**Yellowbelly sliders**			
Disorder undiagnosed	2	100.00	34.24–100.00

## Discussion

To the authors’ knowledge, this is the largest published study to date globally to report demography, disorders and mortality within chelonia under primary veterinary care. With records on 2,040 chelonia assessed, this information provides novel insights into the typical caseloads seen by veterinary clinicians in general practice.

This study has revealed that the most common tortoise species recorded in the UK is the Hermann’s tortoise, and the most common terrapin genus is the musk turtle. This corresponds with the results of the 2023 Federation of British Herpetologists survey [5]. With 58.43% patients not having their species recorded, it is evident there is either a widespread lack of knowledge of the different species of chelonia or that veterinary practices are simply not recording this detail. Given that species information is more commonly recorded for other exotic species within VetCompass studies, for example 99.82% of hamsters had their species recorded, this suggests that it is more likely that veterinary teams and potentially owners are unaware of the chelonian species under their care as opposed to choosing not to record the species [27]. Lack of awareness of what species are being treated, however, puts their veterinary advice at risk of not only being incorrect but also potentially dangerous. For example, the Horsfield’s tortoise naturally hibernates whereas the Sulcata tortoise never hibernates in the wild [28]. Inability to distinguish between these two species could mean that veterinary advice on hibernation could be incorrect. Good awareness of the most common species presenting to veterinarians allows veterinary teams to identify chelonian species more accurately and therefore improve their veterinary care.

Despite 43 patients being recorded as turtles using the vernacular term, no turtle species were recorded. This likely corresponds with the differences in terms used around the globe as explained above. Based on both their size and location, sea turtles are unlikely, although not impossible, to present to primary care veterinarians in the UK. Hence, many of the “turtles” recorded in the current study were likely to have been misclassified tortoises or terrapins.

Of the 2,040 chelonia, 88.53% of patients had sex recorded. Accurately identifying the sex of chelonians can be challenging due to their lack of external genitalia. Techniques for accurately sexing chelonians include endoscopy and hormonal assays, but subjective features such as the curvature of the plastron and tail length are often used instead in clinical practice [29]. However, these features develop dimorphically over time so the sex status recorded using these methods may be poorly reliable, particularly in younger chelonia which is relevant as the median age of the current study population was 9.31 years.

The low median age of chelonia in the current study may simply reflect a truly young population, although this seems unlikely as tortoises have been kept in the UK for many decades [30]. Alternatively, the young age of the chelonian population here under primary veterinary care may reflect selection bias whereby older individuals are not perceived by their owners as requiring veterinary care perhaps because they are often kept outside and may not be closely observed. Such tortoises may have died without any veterinary involvement which would also explain the low median age of death of only 7.32 years. Finally, as stated earlier, it can be extremely challenging to accurately age tortoises and hence the age recorded in the clinical records may be unreliable and provide a minimum estimate of age rather than a true estimate.

The top three recorded disorders at precise-level of diagnostic precision across all three vernacular groups of chelonians were beak abnormality, overgrown nails and shell abnormality. This differs from a 1979 study which reported gastrointestinal helminthiasis, necrotic stomatitis and hypovitaminosis as the top three disorders in tortoises in the UK [14]. The top three recorded disorders at precise-level in the current study all have externally visible signs that require limited further investigations to accurately define. However, it must be considered that these beak, nail and shell disorders can also be associated with systemic disease. It is important to note that these beak, nail and shell disorders are often linked to inappropriate husbandry, highlighting the importance of veterinarians providing evidence-based husbandry advice.

Regarding grouped-level disorders, parasite infestation was reported in 5.34% of the chelonian population. Although not directly comparable due to the differing study design, this is lower than a previous study which reported a 49% prevalent gastrointestinal parasitic burden in tortoises [13]. In the present study, not all chelonia had faecal assessments to assess for the presence of gastrointestinal parasites, whereas the other study did, and hence the current results may underestimate the true levels of parasite infestation.

Both the precise and grouped-level disorders represented quite broad clinical terms, but this represents the terms recorded within the EHRs. When compared to similar studies in other species more precise terms were used in other studies [21]. This suggests that limited clinical workup of disorders in chelonia is common under UK primary veterinary care. Many reasons could explain this, such as financial constraints; further research is needed to better understand what factors may limit diagnostic workup.

Nearly 1 in 10 of the study sample were recorded as having died during the study period (9.90%). The relatively young median age at death of 7.32 years is concerning, although this may just reflect the young population that are presented to primary veterinary care. In reality, the true median age of death of the overall owned chelonian population could be considerably higher if owners selectively do not present their older tortoises for veterinary care. The current result that cause of death was not recorded in 44.55% of deaths is also concerning. Following disorder undiagnosed, dog bite was the second leading cause of mortality. The risk of this form of trauma can be reduced with appropriate husbandry education to owners from veterinarians.

Some of the disorders recorded as causes of death such as azotaemia and abdominal disease are not consistent with reptile pathology. Due to the unique chelonian anatomy and physiology, these terms are technically inappropriate as diagnoses in reptiles. Regardless of appropriateness, the current study extracted the most refined terms recorded in the EHR but this could suggest that many clinicians are poorly aware of the differences between reptile and non-reptile disorder terms and hence further education in this field may be prudent, ideally beginning at veterinary schools.

This study highlights the importance of ensuring primary care veterinarians feel well equipped to recognise the most commonly encountered chelonia species and how to identify, diagnose and treat their disorders. As an extension of this, focus and resources could be placed on deeper investigations into aetiologies behind the commonly encountered disorders in order to reduce their frequency and improve welfare and ultimately ensure these animals survive past puberty.

This study has limitations as previously reported in similar studies of other species [27]. A major limitation if the results of the current study are being used to understand true aetiological disorder patterns is the limited clinical work-ups undertaken for chelonia under primary veterinary care that results in many primary care final diagnoses being poorly specific, e.g., many recorded disorder terms were clinical signs rather than formal biomedical diagnostic terms. Limited familiarity by clinicians with chelonia may have contributed to this phenomenon. However, an aim of the current paper was to report an accurate picture of the clinical care delivered to chelonia under primary veterinary care in the UK and to this end, issues around non-specific diagnostic terms were not a limitation but were an accurate reflection of a true clinical picture. Were a similar study to be conducted in a zoological setting it would be interesting to see if similar issues were present. To the authors’ knowledge no similar studies have been conducted.

**Key recommendations:**

- Prioritise veterinary education on chelonian species identification to ensure correct species identification.
- Prioritise veterinary education, specifically husbandry, covering the most frequently encountered species which are Hermann’s tortoise, Horsfield’s tortoise, Mediterranean spur-thighed tortoise, musk turtles and yellowbelly sliders.
- Ensure veterinary education equips veterinary teams to correctly sex the most frequently encountered species.
- Conduct further research into recorded disorders and specifically causes of these disorders, ultimately to improve health of chelonians.

## Conclusion

This is the largest study to date using primary-care veterinary data that reports on the common species, disorders and mortality recorded by veterinarians on UK chelonia. The findings highlight scope for further education of veterinary teams on chelonian medicine and species recognition to increase clinical confidence in recognising and effectively treating these patients.
